# Giving Bad News: A Qualitative Research Exploration

**DOI:** 10.5812/ircmj.8197

**Published:** 2014-06-05

**Authors:** Fereshteh Aein, Masoumeh Delaram

**Affiliations:** 1 Nursing Faculty, Shahrekord University of Medical Sciences, Shahrekord, IR Iran

**Keywords:** Truth Disclosure, Neoplasms, Mothers, Child, Qualitative Research

## Abstract

**Background:**

The manner in which healthcare professionals deliver bad news affects the way it is received, interpreted, understood, and dealt with. Despite the fact that clinicians are responsible for breaking bad news, it has been shown that they lack skills necessary to perform this task.

**Objectives:**

The purpose of this study was to explore Iranian mothers’ experiences to receive bad news about their children cancer and to summarize suggestions for improving delivering bad news by healthcare providers.

**Materials and Methods:**

A qualitative approach using content analysis was adopted. Semi-structured interviews were conducted with 14 mothers from two pediatric hospitals in Iran.

**Results:**

Five major categories emerged from the data analysis, including dumping information, shock and upset, emotional work, burden of delivering bad news to the family members, and a room for multidisciplinary approach.

**Conclusions:**

Effective communication of healthcare team with mothers is required during breaking bad news. Using multidisciplinary approaches to prevent harmful reactions and providing appropriate support are recommended.

## 1. Background

Over the course of a career, a busy clinician may break bad news to families many times. In a survey regarding breaking bad news, 60% of participants indicated that they broke bad news to patients or their families 5 to 20 times per month and another 14% more than 20 times per month (1).

Delivery of bad news is always an unpleasant but a necessary part of medicine. Breaking bad news is a difficult and uncomfortable process, both for recipient and giver (2). In the case of pediatric cancer, how bad news is delivered can have a significant impact on mothers’ perspectives of illness, their long-term relationships with clinicians and both parent and provider satisfaction (3). The time immediately after the diagnosis has been perceived as the most stressful period, and leads to the greatest loss of quality of life in mothers. In many cases, several years since the bad news encounter, these encounters were remembered down to the smallest details and strong emotional responses were evoked even after a significant period of time (1).

The literature suggests that breaking bad news is often not performed effectively and medical staff lack skills when speaking to recipients (4, 5). This may be the reason why it is very often performed so badly. Effective communication of bad news is a key clinical skill, which clinicians must possess. The initial diagnosis is only the first step in establishing a positive relationship between family and medical team (4, 6). Approaching a family with bad news, but without an appropriate plan to present the information in a structured manner, is almost a guarantee of greater emotional pain and disruption for the recipients of the news (7, 8).

The importance of breaking bad news in the right way cannot be underestimated and is reiterated throughout the literature. Research has shown that if bad news is communicated badly, it can cause confusion, long lasting distress, and resentment; if performed well, it can assist understanding, acceptance, and adjustment (7, 9-11). Despite the fact that physicians are responsible for delivering bad news, there is currently no formal training on how to deliver bad news provided to medical students and resident physicians in Iran.

A number of studies have also shown that medical teams have trouble to deliver bad news. Lack of skills has been reported as the main cause for physicians’ avoidance of this task (6, 12). Many physicians find these interactions stressful, and in the absence of effective training, they may adopt inappropriate ways of delivering bad news and coping with the emotional fall-out (2, 6, 8). Mothers also repeatedly stated the importance of the formulation of a plan for the future (13). Actually, training physicians to do the task more effectively would be beneficial for them as well as families, but this training needs to be based on scientific educational principles, informed by evidence, and assessed and monitored adequately (8).

Moreover, there are considerable differences in the way physicians break bad news between countries worldwide. Physicians sometimes find it hard to step back and take into account the spectrum of physical, socio-cultural, occupational, and emotional aspects that may affect what information is classified as bad news for recipient. In studies of physicians’ views on breaking bad news from 1993 onwards (1, 2, 6), some disparities were found between physicians in opinions about truthful disclosure, stress experienced when giving bad news, and desire for more training (14).

Research findings emphasized that to achieve the goal of building a therapeutic relationship, a method needs to be presented that would give practitioners the tool to develop a trusting relationship with a previous stranger (8, 13, 15-18). Therefore, understanding what is important to recipients when sad or upsetting news is given can help physicians redefine how this task is best done. Although many personal and anecdotal reports have been published in medical and lay press (6), no researcher has systematically assessed how Iranian mothers feel immediately after receiving bad news.

## 2. Objectives

The aim of this study was to determine how mothers in Iran recall the initial discussion regarding a diagnosis of cancer and to identify recommendations on improving the initial discussion they have with clinicians.

## 3. Materials and Methods

A qualitative approach using content analysis was adopted for this study to facilitate a rich description of individual experiences and perceptions of receiving bad news. Data collection compromised unstructured interviews with 14 mothers who were recruited from two large central pediatric hospitals in Tehran, the capital city of Iran, from May to August 2010. Those hospitals take referrals mainly from across the country. Ethical approval was obtained from the ethical committees of hospitals.

The ethical issues in this study involved the assurance of confidentiality and autonomy for the participants. All participants were informed about the purposes and methods of the study. They were also informed that participation in the study is voluntary, so they could refuse to participate or withdraw from the study at any time. Moreover, the participants were reassured that their responses would be kept confidential and their identities would not be revealed in research reports and publications of the study. Lastly, the participants who agreed to participate in the study were asked to sign a written consent.

All interviews were performed by one female interviewer and were audiotaped with the mothers’ consent. The interviewer received formal qualitative curriculum training and had clinical practicum teaching experience of 5 years, which would help the participant enter the interview situation and build a trustworthy relationship. At the interview, the main questions explored the mothers’ experiences when receiving bad news to know their child’s cancer. A semi-structured interview was used. It was composed of four open-ended questions (interview guide) as follows: Who told you that your child has cancer? Please describe the situation and the manner in which the information was given? What were your feelings at the time? And if you had any suggestions to improve the initial discussion with clinicians. The questions served as prompts if the case arose. No prejudices or personal opinions were involved in the interview process, and semi-structured guidelines were adopted to guide interviewees to express their experiences as far as possible. The participants were asked to avoid mentioning specific physicians' names and to discuss their experiences in general terms. Where this occurred, the names were deleted from the transcript. The mothers were recruited by purposeful sampling with the maximum variance of sampling to achieve variation in children's age and type of cancer as well as ethnic groups. Inclusion criteria were having a child with cancer and the maximum time passed after the diagnosis was less than 6 months. An effort was made to recruit a heterogeneous population of mothers, but the study group was not intended to be a representative sample. Mothers were interviewed individually at a time convenient to them during quiet periods in day in a quiet room nearby. Interviews lasted between 50 and 100 min. The participants’ selection, data collection, and data analysis continued until data saturation occurred and a rich description of experiences was obtained. The data collection ceased after 14 interviews, as after 12 interviews, it was clear that no new concepts had emerged. Confirmability, credibility, dependability and transferability were used to assure various aspects of trustworthiness according to Lincoln and Guba (1985) (19). For conformability, the bracketing process was put aside assumptions and biases that were possessed by the researchers before data collection. To assure credibility, we used the maximum variance of sampling, peer debriefing or reviewing of the data, codes and themes by a coresearcher, and member checking of the findings by research participants. Focusing on the research objectives and trying to question the same areas for all the participants was used by researchers during the study to assure dependability. Recruiting participants with the maximum variance of sampling helped transferability of the results. Nevertheless, generalizability is nor a claim neither a primary concern of qualitative research. 

### 3.1. Data Analysis

Content analysis was conducted to analyze data (20). We used five key stages to qualitative data analysis involved in "Framework". In the first stage (familiarization), we transcribed the data and read each interview through several times to gain a sense of content. The second stage (identifying a thematic framework) involved dividing the text into meaning units. The condensed meaning units were abstracted and labeled with a code, which constitute the manifest content.

In the third stage (indexing), we compared the various codes based on differences and similarities and sorted them into sub-categories and categories and collated all the relevant coded into data extracts within the identified categories.

In the fourth stage (charting), we read all the collated extracts for each category and considered whether they appeared to form a coherent pattern. Then, we considered the validity of individual categories in relation to the dataset and whether our candidate categories “accurately” reflected the meaning evident in the dataset as a whole. Two researchers independently examined the data for categories. In the fifth stage (mapping and interpretation), we defined and further refined the categories.

## 4. Results

In total, 14 mothers aged 29 to 48 years old participated in the study. We conducted the interviews in the hospital during children's hospitalization. We included their mothers only because their fathers were not allowed to stay with the child in hospital due to cultural and organizational limitations so we could not access to fathers for interview sessions. The categories identified following content analysis of the interviews were presented under five major categories that illustrate a process of receiving bad news and reacting to them:

(i) dumping information,
(ii) shock and upset;
(iii) emotional work,
(iv) burden of delivering bad news to family members,
(v) a room for multidisciplinary approach.

### 4.1. Dumping Information

This category included three subcategories: dumping information, no assessment of the recipient’s preparedness, and lack of empathy. The most prominent subcategory was the “dumping information given”. This was mentioned by all mothers interviewed. Although most mothers stated that before they were told directly that the diagnosis was cancer, they found out the diagnosis through indirectly listening to medical team rounds during the time awaiting the diagnosis, after the cancer definitely diagnosed, dumping the reality of diagnosis by the physician perceived as a terrible experience for most mothers. Another subcategory was “no assessment of the recipient’s preparedness". Our participants gave numerous quotations of medical professional’s bluntness of information without first checking if the person is prepared to hear it.

Another repeating subcategory was “lack of empathy”. There were moments in which absence of support was most acutely experienced. All commented on physicians or residents who behaved without empathy. Negative comments were conveyed by characterizing physicians as ‘rough’ and ‘aggressive’ while mothers needed physicians to be ‘considerate’, ‘confidence-inspiring’ ‘calm and objective’, ‘smooth’, ‘sympathetical’, ‘caring’’, and ‘understanding’.

Mothers believed that physicians should greet mothers politely before consultation and should avoid disclosing the bad news at the first time. He or she should warn the mothers that bad news was about to be disclosed, so that they could prepare for it. Mothers repeatedly stated the importance of the formulation of a plan for the future.

### 4.2. Shock and Upset

Emotions experienced by mothers when breaking bad news were predominantly shock and upset. The bluntness of information caused a sense of loss of control and evoked powerful emotions of upset and shock. Almost all mothers expressed extreme dissatisfaction with the process. The reaction to how they were told ranged from ‘‘extremely rude’’ to ‘‘shocked by the way the information was delivered despite how hard it was to hear”.

One mother stated that ‘My husband and I were severely shocked by unexpected bad news when physician did not warn us about it. He talked about my child diagnosis without any consideration of our feelings, He was so blunt'. Only one mother was satisfied with bluntness of delivering bad news by physician.

The main factor that played an important role in being shocked and upset was the preconception of mothers about the word “cancer”. Mothers seemed to enter a fog after the word “cancer” was mentioned. For Iranians, cancer is seen as the point of no return and no cure. Three mothers felt devastated by the news having gone through childhood fear of “cancer”. Though mothers preferred to be given the bad news clearly and honestly, they preferred not to use the word ‘cancer’ or “chemotherapy” repeatedly. They wished physicians to choose words carefully and use euphemisms appropriately for example “serious disease” instead of “cancer” or “severe and long-term treatment” instead of “chemotherapy”.

### 4.3. Emotional Work

In reacting to shock and upset that mothers experienced when facing bad news without any preparation to give them some form of control, they went through two stages to cope with the situation:

1. emotional reactions; and
2. using hope as an adaptive force. Emotional reactions such as crying and screaming were the first reactions of most mothers to being shocked by hearing bad news.

Most mothers needed to vocalize their pain by crying. Still, other mothers might remain stoic and silent or might need to intellectualize the process. One mother cited that “Cancer was the most difficult moment. The first tears were then”. Another mother stated that ‘I became confused and shocked first. My husband screamed in the way to home but I began to cry loudly when I got home after keeping silent for a while’. Some other mothers needed to be alone after hearing bad news to cry and vent their emotions. One mother stated:

“I was shocked to hear the news and could not tell anything. I needed to be alone to cry. I still need a private room to cry and vent my emotions”.

Hope as another coping strategy was used by mothers to deal with the situation. This was invaluable in its ability to serve as an adaptive force in the process of hearing and coping with bad news. All mothers became more dependent on their religious faith. Prayer was very helpful in assisting these mothers to cope with the situation. They commonly used prayer and they mostly stated that though they preferred to be given bad news clearly and honestly, after disclosing the bad news, physicians should use some supportive expressions to relieve mothers’ emotional distress, and to reassure and encourage parents such as; do not worry, I am on your side, life is in the hands of God, rely on God and let us do our best together. My husband and I needed hope and physician should not kill our hope’.

It was revealed that mothers did not want to focus on the prognosis of disease. Moreover, there were many comments on simultaneously being given information that would give mother's hope. A mother stated that ‘I hope that my physician would consider my feelings. You cannot imagine how would be when a physician tells the parents that their child has cancer; it is a terrible experience. I never want to remember that moment. He spoke about my child disease as if there was no hope”.

### 4.4. Burdon of Delivering Bad News to Other Family Members

One challenge mothers encountered after hearing bad news was how to inform other family members. All mothers complained that the physician did not devote time to tell the reality of diagnosis to other family members. Dumping information to one of mothers, mostly mothers without including other family members, put the burden of delivering bad news to other family members on the mother’s shoulders. It was a difficult job for mothers. Participants also preferred to be told the bad news in the presence of other family members. One mother stated that “My mother was sitting over there. When I left the physician office, she was awaiting for my answer. Can you imagine? I myself was shocked and I could not tell her anything. She asked me but I was not able to tell the truth”.

Another mother cited that “I hope that my physician would consider my extended family as well as me. I myself needed emotional support but the responsibility of telling news to my family was horrible. I never can do that job”.

While mothers who were dissatisfied about the way of delivering bad news, they expected physicians to use their relatives to help telling the diagnosis to them, other mothers preferred to be told the diagnosis directly by health professionals. One mother stated that “I heard that physicians first told the bad news to relatives not to mothers directly. My husband and I were severely shocked by unexpected bad news when physician told us directly and suddenly.” Another mother cited that “I prefer to be aware of the diagnosis of my child directly by the physician. I do not like to hear the bad news from my family or relatives.”

It is apparent from the data that mothers’ reaction did not depend on the child age but telling the diagnosis to the elder child was another dilemma which mothers were faced. Mothers whose child was of school age or older commented that they would have preferred the child not to be told the diagnosis and they actively tried to hide the diagnosis from the child, though mothers thought that most children suspected the diagnosis but never asked their mothers about it. One mother said that “I did not know what I should tell my child. My child asked me about the reason “Why I have to go to hospital repeatedly? Why my sister and my cousins are healthy but I am always ill? I really did not know what should be my answer to my child but I really did not want to tell the diagnosis to him. I tried to hide the truth from the child”.

### 4.5. A Room for Multidisciplinary Approach

Finally, parents provided suggestions for improving the process of diagnosis, including communication at diagnosis and environment where diagnosis was given. Mothers emphasized that another medical staff member familiar with mothers and children should be present at the time of delivering bad news. All mothers mentioned that in their opinion, a team of professionals should deliver bad news. Sufficient time should be set aside to ensure that all necessary information could be communicated and mothers could ask questions or vent their emotions. Regarding treatment, most participants preferred to know the latest treatment options, availability of treatments, future treatment plan, and adverse effects and risks of treatment. A mother stated that “I did not know what happen to my child in the future. If I knew the next steps of treatment, I prepared myself to cope with it. I think there is a need for a nurse knowing my child and a psychologist beside a physician at the time of delivering diagnosis to mothers. If they were present during the consultation, I could consult with them about what was explained by the physician and anything that was not understood about treatments, as well as receiving emotional support from them”.

## 5. Discussion

Delivering bad and difficult news would always be an unpleasant but necessary part of medicine. Our findings discovered five major categories that illustrate a process of receiving bad news and reacting to them including dumping information, shock and upset, emotional work, burden of delivering bad news to other family members, and a room for multidisciplinary approach. Overall, our study revealed that mothers remembered the disclosure well. Most comments by mothers when asked how they felt about the communication of diagnosis were negative. Few studies reported a high level of satisfaction with the way mothers were told a diagnosis. Similar to the findings of one previous study in the UK (21), our findings showed that both the process of delivery of bad news and the content itself have a profound impact on mothers’ emotional reactions.

Dumping information by physicians without showing empathy, was the first term repeatedly mentioned by mothers. This is similar to the straight-to-the-point manner mentioned by Gao as a mentally devastating manner to all recipients (2). Shaw et al. also reported BLUNT style as delivering bad news without preamble. It occurs when physicians broke news almost immediately while there was little or no attempt to place the news in context and no indications of bad news were provided prior to the news delivery (6). This behavior by health care professionals, which was also reported in previous studies, may root in difficulties of person in delivering the news (5, 13, 22). Mothers in our study experienced a state of shock and upset at the diagnosis, which is international reactions in recipients of bad news (13, 23-25). This is when tears are expressed. Guidelines and breaking bad news (BBN) programs recommend inclusion of warning shots in the news delivery, as forewarning reportedly reduces shock and facilitates emotional work (6). Moreover, according to Kubler-Ross, as the impact of diagnosis is assimilated by recipients, health-care staff should anticipate a range of grief responses as they begin to express their emotions (25). Acknowledging and even encouraging the expression of sadness is invaluable (16). Mothers in our study noted a need for professionals to consider their feelings, but they did not expect them to be overly optimistic about the expected outcome of the interventions. There is a general consensus in medical literature that full disclosure empathy and honesty are required when delivering bad news (6).

Another term mentioned by mothers in our study was terrible meaning of “Cancer” and “Chemotherapy”. As previously demonstrated (8), how bad and sad information is perceived depends on previous experiences. Similar to Parker’s study, in Iranian culture, terms such as “cancer” or “chemotherapy” as reminders of life-threatening diseases are horrible. Though mothers preferred to receive bad news frankly, they preferred not to use the word ‘cancer’ or “chemotherapy” repeatedly, and expect physician to choose words vigilantly and appropriately (26).

Hope was used as an adaptive force by mothers in our study. As our findings showed, reality is accepted soon, at least in a way that allows logical decisions to be made about the child’s illness and the potential treatment. However, instilling realistic hope can be invaluable to mothers. In our study, mothers repeatedly mentioned that hope was worthwhile to provide an adaptive force in the initial process of hearing and coping with bad news, and this was reported before (8). Similarly, Scrimin et al. reported that hope might contribute to mothers’ abilities to identify their strategies in coping with pediatric cancer experiences. These findings confirm the statement that hope appears to be central to mothers who have children with cancer (27). Kylma and Juvakka also suggested that parental hope is integral to help children and families cope with the cancer experience. In our sample, with tragic news, almost all mothers became more dependent on their religious faith. As all our participants were Muslim, It was a strong coping strategy, which helped mothers reserve hope (28). Similarly, Schubert and Chambers believed that encouraging mothers to pray was very helpful in assisting them to cope (16).

Some maternal suggestions for improving the process of diagnosis were also identified in this study. Delivering bad news by a multidisciplinary team including a nurse and a psychologist was one of these suggestions. While many guidelines around that topic focus on multidisciplinary work, others pursue unidisciplinary training. There is little evidence as to which is the most effective, but despite this uncertainty, it is clear that every health-care professional should have training in this field (29).

Burden of delivering bad news to other family members was a concern mentioned by mothers in our study. According to our findings, some mothers preferred to deliver bad news to her companion, for example, her husband or other significant relatives. As discussed by Street and Gordon, families and friends can be a valuable source of support, help, and information as adult patients cope with diagnosis of cancer, its treatment, and recovery (30). Our findings indicated that companions would play a helpful role in delivering bad news to mothers as well but it was not acknowledged by all mothers.

On another side, an interesting finding not well documented in the literature is whether it is appropriate to include child in the initial disclosure. As demonstrated earlier by Parsons et al. mothers stated that they would prefer not to tell their children about the diagnosis and they actively tried to hide the diagnosis from the child, though mothers thought that most of children suspected the diagnosis (31). Other studies showed that the child is informed about a cancer diagnosis by physician in the USA (13), but in Japanese mothers, telling children is still not accepted (32). Consequently, cultural willingness should be taken into consideration by health professionals.

### 5.1. Implications for Practice

Because bad news may significantly change a family view for the future, particularly when it involves an oncologic diagnosis, it is crucial to do it well.

Who should be involved in delivering bad news? Communication of the initial diagnosis of any life threatening illness can set the tone for long-term psychological adjustments for mothers. Our findings indicated that clinicians need to spend more time to know mothers’ willingness before deciding about delivering bad news directly to mothers or child or by getting help from family relatives. Knowing mothers’ decision whether they need any support from other persons, like other family members, is important. Identifying family members and including them when the news is delivered is a key factor in facilitating the process of giving bad news. Iranian families in our study desired to protect their child from bad news. Considering this issue is important. Moreover, delivering bad news by a multidisciplinary team including a nurse and a psychologist was another suggestion from mothers. Staffs who have developed a connection with family could be enlisted as sources of support. From the mother's perspective, the best way to be informed is through direct face-to-face communication from physician who knows the family best.

How to deliver bad news? To deliver bad news well, research to date has suggested that a therapeutic relationship must first be built. Then, bad news would be shared with a family in its best possible way. It must be kept in mind that these families are about to experience a significant loss of control in their lives. Anything that can be done to give them back a bit of control would be beneficial to their coping with the situation. Creating a setting where the family has some control is the first step in constructing a productive encounter. However, guidelines advise to find a suitable place to disclose the news and dose information to reduce the shock experience of recipients (33, 34). Thereby, families would be able to hear bad news and set the stage for constructive adaptation to their new life situation. 

Building rapport involves attention to the emotional climate of the family and being in tune with their needs. The most important elements of building a foundation for a therapeutic relationship are empathy and compassion. Communicating with empathy and compassion goes a long way in making this task easier and more effective. Most mothers desire frank, direct presentation of the diagnosis, although conveyed with compassion. Though honesty and confrontation are necessary in delivering bad news, they must be tempered with patience and empathy. Current BBN guidelines recommend the use of a direct approach when BBN (8).

Moreover, the findings of the present study together with other studies clearly indicated that hope is crucially important to mothers in their maternal work and coping with a child’s illness cancer. This is a time where instilling realistic hope can be invaluable to family. Being up front with a family while allowing for optimism can be a challenge. A family should never be deceived, but giving realistic assurance that any possible management would be performed and that the family would not be abandoned in the process is an essential component of preparing them for the future aspects of child’s illness. It is important for mothers to be guided through the treatment process and to be aware exactly of what is supposed to happen in the near future.

### 5.2. Limitations

While this study was performed with only 14 mothers, it provided a good description about the phenomenon of breaking bad news in clinical settings. The flexibility and other strengths of the qualitative methodology made it possible to describe mothers’ experiences. Results from this study can be used to refine existing guidelines on how to train health care professionals in breaking bad news to mothers. However, our results stem from a qualitative study and bringing the study implications into practice to the ‘‘real’’ world requires additional studies; nonetheless, our results can be examined in a more applied setting. In addition, as this is a qualitative study, the study group is in no way a representative sample. Therefore, the results of this study are predominantly representative of the group of mothers who participated in this study and cannot be generalized to all mothers of children with cancer. However, generalizability was neither the aim nor claim of this study.
